# Following a Long-Distance Classical Race the Whole-Body Kinematics of Double Poling by Elite Cross-Country Skiers Are Altered

**DOI:** 10.3389/fphys.2018.00978

**Published:** 2018-07-25

**Authors:** Chiara Zoppirolli, Lorenzo Bortolan, Federico Stella, Gennaro Boccia, Hans-Christer Holmberg, Federico Schena, Barbara Pellegrini

**Affiliations:** ^1^CeRiSM (Research Center Sport Mountain and Health), Rovereto, Italy; ^2^Department of Neuroscience, Biomedicine and Movement, University of Verona, Verona, Italy; ^3^NeuroMuscularFunction Research Group, Department of Medical Sciences, School of Exercise and Sport Sciences, University of Turin, Turin, Italy; ^4^Swedish Winter Sports Research Centre, Department of Health Sciences, Mid Sweden University, Östersund, Sweden; ^5^School of Sport Sciences, UiT The Arctic University of Norway, Tromsø, Norway

**Keywords:** whole-body kinematics, fatigue, marathon, cross-country skiing, centre of mass, stretch-shortening cycle

## Abstract

**Introduction:** Although short-term (approximately 10-min) fatiguing DP has been reported not to alter the joint kinematics or displacement of the centre of mass (COM) of high-level skiers, we hypothesize that prolonged DP does change these kinematics, since muscular strength is impaired following endurance events lasting longer than 2 h.

**Methods:** During the 58-km Marcialonga race in 2017, the fastest 15 male skiers were videofilmed (100 fps, FHD resolution in the sagittal plane) on two 20-m sections (inclines: 0.7 ± 0.1°) 48 km apart (i.e., 7 and 55 km from the start), approximating 50- km Olympic races. The cameras were positioned perpendicular to and about 40 m from the middle of each section and spatial dimensions adjusted for each individual track skied. Pole and joint kinematics, as well as displacement of the COM during two DP cycles were assessed.

**Results:** The 10 skiers who fulfilled our inclusion criteria finished the race in 2 h 09 min 19 s ± 28 s. Displacements of the joints and COM were comparable to previous observations on skiers roller skiing on a flat treadmill at similar speeds in the laboratory. 55 km after the start, cycle velocity and length were lower (*P* < 0.001 and *P* = 0.002, respectively) and the angular range of elbow joint flexion during the initial part of the poling phase reduced, while shoulder angle was greater during the first 35% of the DP cycle (all *P* < 0.05). Moreover, the ankle angle was increased and forward displacement of the COM reduced during the first 80% of the cycle.

**Conclusion:** Prolonged DP reduced the forward displacement of the COM and altered arm kinematics during the early poling phase. The inefficient utilization of COM observed after 2 h of competition together with potential impairment of the stretch-shortening of arm extensor muscles probably attenuated generation of poling force. To minimize these effects of fatigue, elite skiers should focus on maintaining optimal elbow and ankle kinematics and an effective forward lean during the propulsive phase of DP.

## Introduction

Technical skills are extremely important to the success of elite cross-country skiers and, consequently, constitute an integral part of their training schedule. Sport-specific activities, such us skiing, roller skiing, and training movement-specific maximal strength, power, core stability and motor control, are key elements of training by world-class cross-country skiers (Sandbakk et al., 2011; Sandbakk and Holmberg, 2014). Indeed, the production of propulsive force is determined not only by muscular strength, but also by the kinematics with which each specific technique is performed. For example, rapid propulsive action with proper timing of force application is related to high-level performance with several cross-country skiing techniques (Stöggl and Müller, 2009; Stöggl et al., 2010). In addition, elevation and forward positioning of the centre of mass (COM) during the beginning of the poling phase of double poling leads to greater propulsive force (Zoppirolli et al., 2015).

Fatigue might alter the kinematics of cross-country skiing, either directly by changing the coordination of movements and/or indirectly by reducing muscle force. A limited number of studies have examined the effects of short-term fatiguing skiing exercises on the whole-body kinematics of double poling, but to our knowledge none have focused on the relationship between fatigue and the whole-body kinematics associated with other cross-country skiing techniques. Between the final spurts of successive bouts of a simulated classical sprint race on-snow, Zory and colleagues observed slight modifications in the joint angular displacement of high-level cross-country skiers while double poling (Zory et al., 2009). Hip flexion was less during the poling phase and hip extension lower at the end of the recovery phase, leading to the hypothesis that fatigue reduces both the contribution of the trunk to propulsion and the effectiveness of the preparation phase. In contrast, our research group found no significant differences in the displacement of joints and the COM while double poling at the same sub-maximal intensity before and after short-term high-intensity fatiguing exercise (Zoppirolli et al., 2016). We therefore proposed that the reduction in the poling force observed in high-level cross-country skiers is due to neuromuscular fatigue rather than any alteration in whole-body kinematics.

Apparently, no investigations on the effects of prolonged cross-country skiing on whole-body kinematics have been performed. However, the influence of neuromuscular fatigue following cross-country skiing marathons on motor drive and/or excitation-contraction coupling has been examined. Millet and Lepers (2004) demonstrated that after such a skating marathon neuromuscular fatigue in knee extensor muscles is primarily of peripheral origin. Boccia’s research group observed both central and peripheral fatigue after a classical cross-country skiing marathon (Boccia et al., 2016). Although the central fatigue in elbow and knee extensor muscles was similar, peripheral fatigue was more pronounced in the former, a difference attributed to the repetitive stretch-shortening of these muscles during double poling (Nicol et al., 2006), as well as to the extensive acidosis likely to be present in upper-limb muscles exercising at high intensity due to the different muscle fiber compositions. Other investigators found that peripheral fatigue is the primary cause of loss of strength and poorer performance following high-intensity cross-country skiing of short duration (Zory et al., 2006, 2011; Zoppirolli et al., 2016).

The aim of the current study was to evaluate the effects of a long-distance classical cross-country skiing race on whole-body kinematics and cycle timing during double poling. For this purpose, the 2-D displacements of joints and the COM of elite cross-country skiers at the beginning and end of a 58-km classical marathon (during which only double poling was used) were determined. Our hypothesis, based primarily on the reduction in muscular strength known to be caused by prolonged cross-country skiing, was that double poling for longer than 2 h reduces both cycle velocity and length, together with alterations in whole-body kinematics, primarily with respect to the displacement of upper-body joints and COM positioning during the poling phase.

## Materials and Methods

### Competition and Experimental Section

The double-poling kinematics of elite skiers who participated in the 44th Marcialonga ski marathon (2017), a classical cross-country ski race held annually in Val di Fiemme (Italy) as part of the FIS ski marathon circuit, were analyzed. The course is usually approximately 70 km in length, but was reduced to 58 km that year due to lack of snow. The course was mainly flat and double poling was the only technique used by the first 50 athletes who finished first. Several weeks before this competition, we inspected the first and last parts of the course for suitable sections for filming. Straight 20-m sections with an incline of <1° and located 7 and 55 km from the starting line were selected. The approximately 48-km distance between these two sections is close to the length of a 50-km Olympic race.

### The Skiers

We analyzed the video recordings of all 50 skiers who crossed the two sections selected first, to ensure inclusion of the first 15 who finished first overall. The identities of the first 10 out of the 15 skiers who met the inclusion criteria described below were unknown to us during the analysis. The general characteristics of these skiers (age, height, weight and FIS points) are presented in the Results.

### Filming

Prior to the race, a full high-definition camera (FZ 200 LUMIX, Panasonic Corp., Osaka, Japan) was positioned perpendicularly to each section to be filmed, approximately 40 m from its midpoint and level relative to the horizontal line. This set-up, in combination with recording at 100 fps and maximal zooming in on the 20 m of interest, were chosen to minimize image distortion in the sagittal plane. In addition, another camera (GoPro Hero, GoPro Inc., San Mateo, CA United States) was positioned, at the beginning of and pointing toward the central portion of each section, to allow recognition of each skier and the track he skied.

### Space Calibration and Environmental Measurements

**FIGURE 1:**
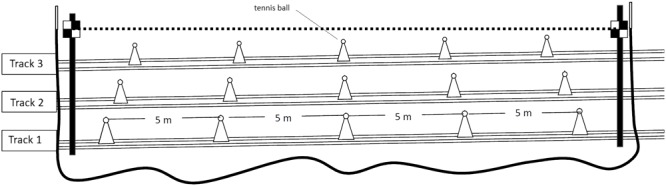
Calibration of the tracks. Cones with a tennis ball on top were placed 5 m apart alongside the middle of each track in the 20-m section. Two 2-m poles (black) were planted near the beginning and end of the section, outside the field of competition. A horizontal line was drawn between the black-and-white reference squares on these poles, ensuring levelness with a custom-made system (a long tube of soft plastic 1 cm in diameter and filled to 80% with alcohol). This calibration was necessary to determine the spatial dimensions of each track skied.

To ensure that the kinematic analysis was as accurate as possible, both filming sections were calibrated carefully before the skiers arrived. The horizontal leveling was checked with a custom-made liquid-level system. Five cones with a tennis ball on top were positioned equal distances apart alongside the three parallel track in each section (**Figure 1**). Thereafter, a short calibration video was recorded. This set-up allowed determination of spatial parameters for each individual track. Everything except the system for assuring the horizontal level was removed following this calibration.

The air and snow temperatures and atmospheric humidity at both sections were measured before the skiers arrived. The weather was sunny, with no wind, and on the first and second sections the snow temperature was -18° and -5°, respectively; the air temperature -10° and +1° and humidity 68 and 68%, respectively. Moreover, snow-ski friction was assessed on the basis of the deceleration (measured employing a photocell with 1-ms resolution) of a skier (not involved in the competition) after accelerating by double poling and then gliding on the track in a crunch position. This skier passed four gates 1, 4 and 1 m apart and deceleration was calculated as the difference in velocity from gate 1 to 2 and from gate 3 to 4, divided by the time required to move from gate 2 to 4. This test was performed on each section by two skiers, both weighing 70 kg and with skis prepared in the same manner. The mean values from three consecutive trials was used to calculate deceleration. Thereafter, snow-ski friction was calculated according to Budde, assuming friction to be the only force acting against the skier’s movement (i.e., neglecting air drag), utilizing the formula:

$$\begin{array}{c} \mu \;=\;a\cdot {\left(g\cdot cos\alpha \right)}^{-1}, \end{array}$$

where *μ* is the snow-ski friction coefficient, *a* deceleration, *g* gravitational acceleration and *α* the incline of the track (Budde and Himes, 2017). We are aware that ski wax will be altered by prolonged skiing and that measurement of ski friction with skies that had been used for approximately 50 km would have provided a more realistic estimate of the snow-ski friction during the second session. However, this was not possible for practical reasons, i.e., the same track could not be skied immediately before the race and there was no other way to accurately reproduce the alterations in the ski wax.

### Inclusion Criteria and Tracking Procedures

Of the 15 male skiers who finished first, we analyzed the first 10 who (a) were completely visible for the entire length of both filming zones (i.e., not obscured by other skiers), (b) completed two DP cycles within each 20-m section, (c) did not change the track or (d) look back (e.g., at opponents) during the filming, and (e) had a cycle velocity on both sections within 2 km⋅h^-1^ of the mean value for the entire group.

**FIGURE 2:**
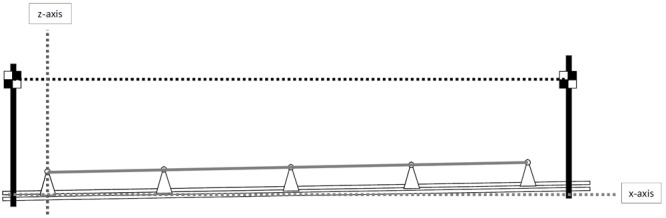
Calibration of each track with the *Tracker* software. The solid gray line joining the center of the tennis balls on the top of the outermost cones was used for calibration (20 m long, in the real situation). The dashed gray lines indicate the bi-dimensional Cartesian plane. The *x*-axis was a line parallel to the horizontal plane (dashed black line). The *z*-axis, perpendicular to the *x*-axis, was provided automatically by the software. The origin of the Cartesian plane was positioned at the base of the first cone. The incline of the track was the incline of the calibration tool relative to the *x*-axis.

All videos recordings and calibration photograms were processed with the *Tracker* software (Tracker 4.11.0 Copyright^©^ 2017 Douglas Brown) ^1^. A 20-m calibration space was defined by a line joining the centers of the tennis balls on top of the first and last cones (**Figure 2**), with a maximal error in the inter-cone distances of 0.05 m (1%), minimizing image distortion. For each track, we defined a bi-dimensional Cartesian plane with its *x*-axis (antero-posterior dimension) parallel to the horizontal plane, *z*-axis (vertical dimension) perpendicular to this same plane and origin at the base of the first cone. The incline of each track was determined as the angle between the *x*-axis and the 20-m calibration line (**Figure 2**). Calibration was performed for each individual track skied, and uploaded on video clips according to the track used by each athletes.

Tracking of each skier was initiated at the time of his first pole plant (PP, i.e., the beginning of the poling phase) after passing the origin of Cartesian plane, and continued until the third PP, i.e., for two complete cycles of double poling. The three PPs and two pole offs (PO, i.e., the end of the poling phase) were identified from the first frame in which the tip of the pole showed no horizontal or vertical movement, or when movement began, respectively. At these time-points both the tip of the pole and another random point near the top of the pole were tracked manually, in order to calculate the pole angle. Semi-automatic tracking (i.e., based on the creation of two recognition zones around the point of interest) was applied to identify the x and z coordinates of the center of rotation of the shoulders, elbows, hips, knees and ankles, as well as the center of the hands and tips of the feet in each frame.

### Parameters and Data Analysis

The horizontal (x) and vertical (z) coordinates of each point, as well as the exact associated time-point, were processed with the MATLAB R2017a software (The MathWorks, Inc., Natick, MA, United States) using a custom-written code. To compensate for the skier’s vertical position relative to the origin, the z-coordinate of each point of interest was corrected for any specific x-coordinate with the formula:

$$\begin{array}{c} z_{corr}\;=\;z-(x\cdot cos\alpha \cdot sin\alpha ) \end{array}$$

where *α* was the track incline relative to the *x*-axis. This correction allowed comparison of the joint and COM displacements of all subjects skiing on different tracks in the two sections.

For each skier, cycle duration, length and frequency were calculated on the basis of the mean time that elapsed and distance traveled between consecutive PPs. Poling duration was the mean time between a PP and the next PO, expressed in both seconds and percentage of the cycle time. The mean pole angles at the three PP and two PO were also calculated.

**FIGURE 3:**
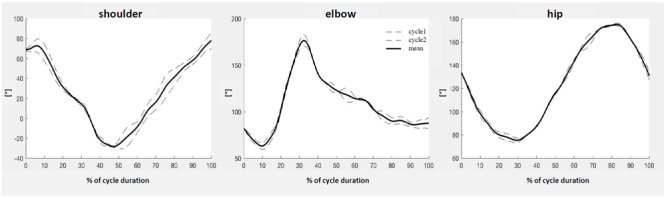
Representative analysis of the kinematics of three of the joints examined. Displacement of the joints and COM during each of the two cycles (dashed lines) was re-sampled at 100 points, in order to calculate the mean cycle displacement for each athlete (solid line).

Starting from the x- and z-coordinates of the point monitored, the angles of the shoulder, elbow, hip, knee, and ankle joints in the sagittal plane during the two DP cycles were calculated. In addition, the vertical displacement of the center of mass (zCOM), as well as its antero-posterior displacement (𝜃COM, calculated as the angle of a line between the medial point of the foot and COM, and the vertical line) were determined as proposed previously (Zoppirolli et al., 2015). All of the data for each cycle were re-sampled at 100 points. Joint and COM displacement data of the two cycles were overwritten in order to control for accuracy and repeatability as well as to compute a mean cycle for each athlete (**Figure 3**).

### Statistical Analyses

**FIGURE 4:**
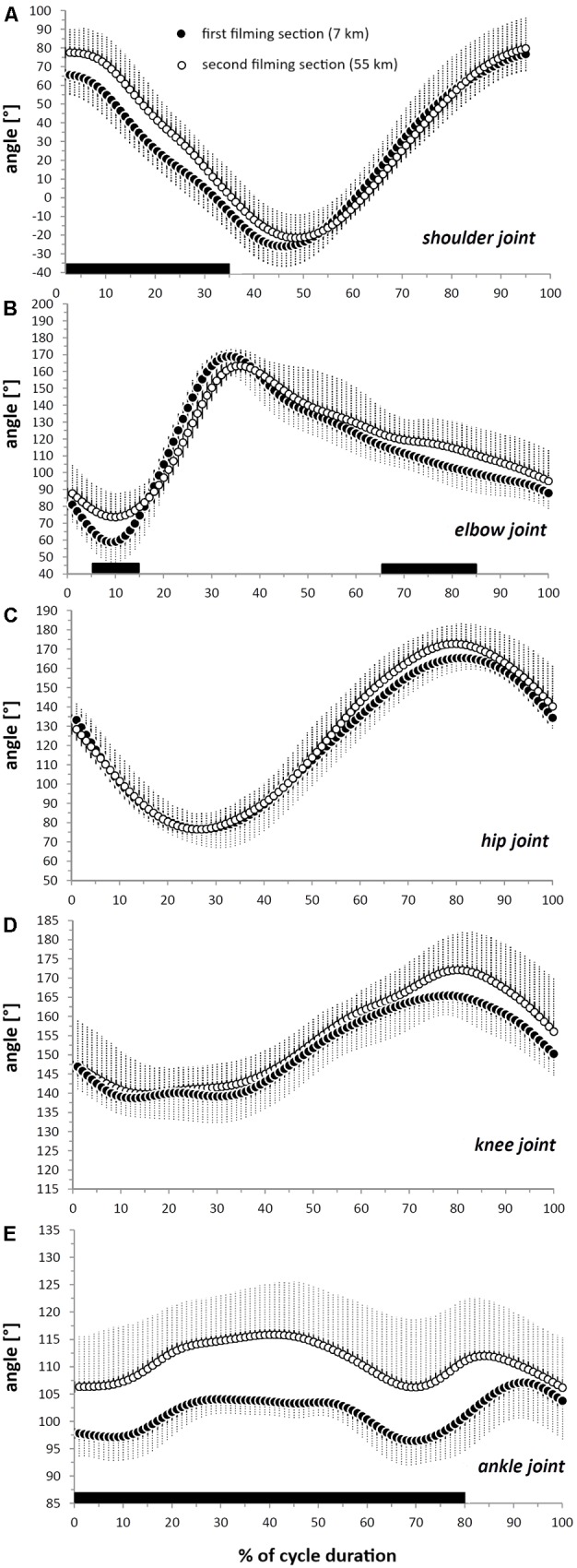
Displacement of the shoulder **(A)**, elbow **(B)**, hip **(C)**, knee **(D)**, and ankle **(E)** joints during an averaged and time-normalized (100 points) cycle of double poling. The black circles show values on the first section (7 km after the start) and the white circles values on the second section (55 km after the start). The horizontal black bars indicate the portions of the cycle during which the difference between these sections was statistically significant (*P* < 0.05).

**FIGURE 5:**
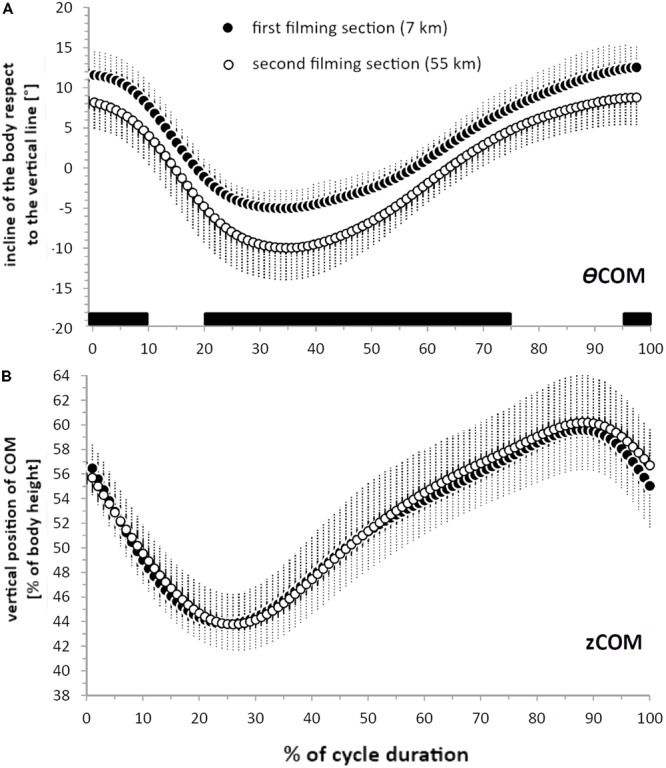
Incline of the body respect to the vertical (𝜃COM) **(A)**, and the vertical position of the centre of mass (zCOM) **(B)** during an averaged and time-normalized (100 points) cycle of double poling. The black circles show values on the first section (7 km after the start) and the white circles values on the second section (55 km after the start). The horizontal black bars indicate the portions of the cycle during which the difference between these sections was statistically significant (*P* < 0.05).

Values are presented as means ± SD. The Shapiro–Wilk test was employed to verify normal distribution of the data concerning cycle time, joint angles and position of the COM at specific time-points in the cycle (i.e., PP and PO). The *Student t-test* was used to evaluate potential differences in these parameters between the two sections (these results are presented in **Tables 2, 3**). Moreover, a more detailed statistical approach was employed to compare joint and COM displacement on the two sections: a two-way ANOVA for repeated measures was performed for each 5% time segment of the cycle, thereby providing a measure of significance for 20 equal segments within the cycle (results presented in **Figures 4, 5**). The IBM SPSS Statistics 22 software (IBM Corp., Armonk, NY, United States) was utilized for these statistical analyses and a *P-value* of less than 0.05 considered statistically significant.

## Results

The 10 skiers who fulfilled the criteria for inclusion (whose anthropometric and performance characteristics are described in **Table 1**) finished the 58-km race within 1 min 18 s after the winner, who finished in 2 h 8 min 36 s. The cycle velocity declined from 24.3 ± 0.9 km⋅h^-1^ on the first section to 22.5 ± 0.8 km⋅h^-1^ on the second (*P* < 0.001, **Table 2**), even though the snow-ski friction coefficient decreased (from 0.054 ± 0.005 and 0.036 ± 0.004, on the first and second sections, respectively). While the cycle frequency (*P* = 0.155) and angle of the pole at PP and PO remained unaltered, both the cycle velocity (-7.9%, *P* < 0.001) and length (-13.1%, *P* = 0.002) decreased and the duty factor rose (+10.1%, *P* = 0.034) (**Table 2**). However, since cycle velocity was not correlated with the mean, minimal or maximal joint angles or position of the COM at PP or PO, we concluded that variations in cycle velocity were not responsible for eventual differences in whole-body kinematics and, therefore, did not include cycle velocity as a covariate in the statistical analyses.

**Table 1:** Anthropometric and performance characteristics of the 10 skiers who fulfilled the criteria for inclusion.

	Mean ± SD of the 10 athletes analyzed
Age (years)	32 ± 6
Height (m)	1.80 ± 0.05
Weight (kg)	74 ± 4
FIS points	139 ± 71

*FIS points are referred to the list relative to the period just before the race.*

**Table 2:** Cycle characteristics, range of motion (ROM) of joints, antero-posterior (𝜃COM) and vertical (*z*COM) displacement of the COM on the first and second sections filmed.

	First section	Second section	*P*-value
Cycle velocity (km⋅h^-1^)	24.3 ± 0.9	22.5 ± 0.8	**0.000**
Cycle duration (s)	1.10 ± 0.09	1.04 ± 0.07	0.153
Cycle frequency (Hz)	0.92 ± 0.07	0.96 ± 0.06	0.155
Cycle length (m)	7.45 ± 0.51	6.58 ± 0.53	**0.002**
Poling time (s)	0.29 ± 0.02	0.30 ± 0.02	0.295
Poling time (% of cycle duration)	26.0 ± 3.1	28.9 ± 2.6	**0.034**
Shoulder ROM (°)	105 ± 14	108.1 ± 17.5	0.699
Elbow ROM (°)	112 ± 14	98 ± 16	**0.045**
Hip ROM (°)	91.3 ± 7.0	99.1 ± 11.9	0.090
Knee ROM (°)	31.5 ± 5.3	35.7 ± 8.6	0.205
Ankle ROM (°)	15.1 ± 4.2	17.3 ± 3.8	0.225
𝜃COM ROM (°)	18.5 ± 3.2	19.5 ± 3.6	0.517
zCOM ROM (% of body height)	16 ± 3	17 ± 2	0.534

*The values presented are means ± SD. The P-values for the differences were determined with Student’s t-test, with values < 0.05 being considered statistically significant. m, meter; s, second; ROM, range of motion; 𝜃COM, incline of the body respect to the vertical line; zCOM, vertical position of the COM.*

The kinematic analysis in the sagittal plane revealed that both the absolute values and ranges of motion (ROM) of the hip and knee joints, as well as the *z*COM displacement did not differ between the two sections (all *P* > 0.05) (**Table 2** and **Figure 4**). In contrast, the angles of the shoulders, elbows, and ankle joints and antero-posterior displacement of the COM were dissimilar at some specific points in the DP cycle. Thus, the shoulder joint had a wider angle during the initial 35% of the cycle time (*P* < 0.050) on the second section, but similar angles for the remainder of the cycle (*P* > 0.050) (**Figure 4**). However, the total shoulder ROM remained the same (*P* > 0.05, **Table 2**).

Moreover, although the angle of the elbow joint at PP (**Table 3**) and during the first 5% of the cycle time (*P* > 0.05, **Figure 4**) was similar on both sections this angle was more extended between 5 and 15% of the cycle (*P* < 0.050) on the second section, when the typical local minimum of this angle was higher (**Table 3**). Consequently, the ROM of the elbow joint during flexion was reduced (from 23 ± 7 to 15 ± 8, *P* = 0.033), as was the overall elbow ROM (**Table 2**). Moreover, the elbow joint was also more extended between 65 and 85% of the cycle time (**Figure 4**, *P* < 0.050) on the second section.

**Table 3:** Minimal (min) and maximal (max) pole and joint angles, as well as displacement of the COM, on the first and second sections filmed, as well as at specific phases of the double poling cycle (PP, pole plant; PO, end of the poling phase).

		First section	Second section	*P*-value
Pole angle	at PP	80 ± 2	79 ± 2	0.378
	at PO	23 ± 2	23 ± 1	0.988
Shoulder angle	at PP	65.7 ± 10.3	77.6 ± 13.2	**0.037**
	at PO	13.2 ± 7.8	19.2 ± 10.5	0.164
	Min	–27.7 ± 8.9	–24.3 ± 11.0	0.456
	Max	77.6 ± 8.8	83.8 ± 13.1	0.230
Elbow angle	at PP	81.0 ± 10.3	87.7 ± 17.2	0.307
	at PO	152.6 ± 8.7	152.9 ± 15.2	0.959
	Min	58.0 ± 11.1	72.5 ± 13.9	**0.019**
	max	171.0 ± 9.0	170.5 ± 13.4	0.929
Hip angle	at PP	133.3 ± 5.4	128.5 ± 14.1	0.325
	at PO	75.9 ± 8.0	76.0 ± 7.8	0.967
	min	75.5 ± 8.1	74.9 ± 7.8	0.874
	max	166.8 ± 5.7	174.0 ± 11.5	0.092
Knee angle	at PP	146.9 ± 5.7	147.0 ± 12.1	0.984
	at PO	139.2 ± 6.8	141.0 ± 5.8	0.529
	min	136.4 ± 6.4	137.4 ± 7.2	0.753
	max	167.9 ± 6.6	173.1 ± 9.3	0.167
Ankle angle	at PP	97.8 ± 4.1	106.3 ± 9.3	**0.017**
	at PO	103.8 ± 2.9	114.8 ± 9.0	**0.002**
	min	93.6 ± 2.6	101.6 ± 10.5	**0.032**
	max	108.7 ± 5.8	118.9 ± 8.6	**0.006**
𝜃COM angle	at PP	11.6 ± 3.2	8.2 ± 3.3	**0.030**
	at PO	–4.1 ± 2.1	–9.2 ± 3.9	**0.002**
	min	–5.4 ± 2.2	–10.1 ± 4.1	**0.004**
	max	13.2 ± 2.9	9.4 ± 3.0	**0.012**
zCOM (% of body height)	at PP	56 ± 2	55 ± 3	0.504
	at PO	44 ± 2	44 ± 3	0.859
	Min	43 ± 2	43 ± 3	0.998
	Max	60 ± 3	60 ± 4	0.693

*All angles are given in degrees and the values presented as means ± SD. The *P*-values for the differences were determined with Student’s t-test, with values < 0.05 being considered statistically significant. 𝜃COM, incline of the body respect to the vertical line; zCOM, vertical position of the COM.*

Furthermore, on the second section the angle of the ankle joint was wider the initial 80% of the cycle duration (*P* < 0.050), but its total ROM was not different (*P* > 0.225, **Table 2**). Although the vertical displacement of the COM did not change (**Figure 5**), the 𝜃COM was less inclined relative to the vertical for most of the cycle on the second section. Thus, there were significant differences in this respect at 0–10, 20–75%, and 95–100% of the cycle (**Figure 5**, *P* < 0.050) whereas its total ROM was unchanged (**Table 2**).

## Discussion

The present findings show that following 50 km of double poling racing, elite cross-country skiers exhibit (a) reduced skiing velocity, (b) an increase in duty cycle, (c) unaltered cycle frequency, (d) changes in shoulder, elbow and ankle kinematics during certain parts of the cycle, as well as (e) reduced displacement of the COM in the forward direction prior to and during the propulsive phase. At the same time, the kinematics of the hips and knees and vertical displacement of the COM remained similar.

### Double Poling Velocity

The snow-ski coefficient of friction was probably lowered by the higher snow and air temperatures on the second section, a reduction presumably experienced by all of the skiers. This coefficient decreases progressively as the air temperature rises from below (around -10°C) to above zero (around + 5°C), irrespective of ski grinding and waxing. Budde and Himes estimated that when skiing on flat terrain, the time required to ski each kilometer increases by approximately 2 s for every 0.001 increase in the coefficient of friction (Budde and Himes, 2017). Since the snow-ski friction coefficient was 0.014 lower on the second section here, skiing velocity would theoretically have been 19% or 28 s per kilometer faster on the second section, rising from 24 to 30 km⋅h^-1^ (assuming the same extent of fatigue). However, the skiing velocity actually fell by 8%, or 12 s per kilometer, apparently due to fatigue. Even though the difference in friction coefficient between the two sections filmed might have been overestimated slightly by our methodology, a recent report on a previous Marcialonga race on the same 58-km track supports our proposal that the decrement in speed is due to fatigue, since neuromuscular alterations in both the arms and legs were observed after that competition (Boccia et al., 2016).

The positive pacing strategy adopted by our skiers is common among competitors in many other endurance sports as well (Abbiss and Laursen, 2008). The 8% reduction in skiing velocity observed here is similar to the small decreases demonstrated previously by elite male cross-country skiers during middle- or long-distance competitions (Carlsson et al., 2016; Losnegard et al., 2016; Welde et al., 2017), as well as with findings on elite runners (Hanley, 2016). This reduction in the second section was due primarily to shorter cycles, since cycle frequency was unaltered.

### Cycle Frequency

The self-selected frequency of movement while, e.g., walking (Bereket, 2005), running (de Ruiter et al., 2014), cycling (Brisswalter et al., 2000), or cross-country skiing (Leirdal et al., 2013) is close to the frequency that minimizes the energetic cost. In particular, Lindinger and Holmberg have shown that double poling at 60 cycles per minute (i.e., 1 Hz, approximately the frequency chosen by our skiers on both sections) is more beneficial in several ways than double poling at 80 or 40 cycles per minute, especially at high velocities (Lindinger and Holmberg, 2011). At 1 Hz, poling force was moderate at all speeds examined (12, 18, and 24 km⋅h^-1^), with lower oxygen consumption, heart rate and blood lactate values at low and moderate velocities, and even minimal values for these parameters at the highest velocity. These authors proposed that utilization of such a frequency at relatively high velocities guarantees minimal poling times, effective generation of poling force, sufficient recovery time for repositioning the body, and efficient muscle perfusion and removal of lactate, as well as an acceptable level of mechanical work and rhythm of breathing.

The effects of long-lasting exercise of different types on movement frequency are controversial. For example, cycling cadence was reported to decline by approximately 10 rpm when exercise continuoused for longer than 1.5 h (Brisswalter et al., 2000; Hansen and Smith, 2009), whereas stride frequency was maintained or decreased only slightly between the initial and final sections of marathon or ultra-marathon running (Hunter and Smith, 2007; Schena et al., 2014; Giovanelli et al., 2017). Less frequent movements may reflect a shift of the power-velocity curve toward the right: a certain amount of power (needed for body propulsion) is exerted with slower contraction in the fatigued than unfatigued state (Jones and Watt, 1971).

Unfortunately, to our knowledge nothing concerning the effects of cross-country skiing for 1 h or more on choice of cycle frequency by elite athletes has yet been published. Recently, Welde et al. (2017) observed no difference in the frequency of double poling or double poling with kick (around 0.92 Hz) by top-level skiers between the first and last laps on the flat section and intermediate incline of a 15-km classical race. In addition, others have found no significant differences in double poling frequency by elite skiers between the final spurt during the first and last bouts of simulated classical sprint races (Zory et al., 2009; Mikkola et al., 2013; Asan Grasaas et al., 2014). In the case of our investigation, cycle frequency remained unchanged throughout the race, probably because highly skilled athletes strive for this, shifting internally their focus of attention to manage a long-duration effort, in order to optimize performance and perhaps minimize the risk of injury (Morgan and Pollock, 1977; Masters and Ogles, 1998; Tenenbaum, 2001).

### Displacement of the Joints and COM

While maintaining the same flexion-extension pattern and range of motion, the ankle joint was approximately 10° more extended for much of the cycle on the second section, possibly indicating a more vertical body, with less forward lean. Indeed, on this same section the incline of the 𝜃COM was approximately 4–5° less during the first 10% of the poling phase, most of the recovery phase and the final 5% of the cycle. This finding highlights once again the importance of the forward lean of the body to exploit gravitational forces during the first part of the poling phase, when peak poling force is attained (Zoppirolli et al., 2015). With double poling, activation of the hip and trunk flexors is higher at faster than slower speeds and also begins significantly earlier in relationship to pole plant, stiffening the core muscles to enable skiers to pole powerfully (Zoppirolli et al., 2017). Here, we demonstrate that prolonged double poling at elevated speed limits the ability to maintain a forward-leaning body during the poling phase, perhaps due to reductions in the strength of the core and abdominal muscles. Accordingly, we propose that the kinematic changes observed in the ankle joint and 𝜃COM are induced by fatigue and designed to lower the stress on core muscles.

In our opinion, another important kinematic modification related to the diminished forward lean of the body is the alteration in the displacement of the elbow joint that occurs during the first part of the poling phase on the second section. The elbow angle at pole plant was the same on both sections, but the minimal angle that typically occurs after 10–12% of the cycle duration (Lindinger et al., 2009; Zoppirolli et al., 2013) was increased significantly on the second section, thus attenuating the range of flexion that precedes the extension phase. Other investigators have suggested that with an initial double poling velocity of 16 km⋅h^-1^, a stretch-shortening cycle occurs in the extensor muscles of the arm immediately after pole plant, during the flexion-extension of the elbow joint (Lindinger et al., 2009; Zoppirolli et al., 2013). Thus, the limited angular range of the elbow joint on the second section probably reflects a reduced capacity of these extensors for sustaining eccentric work. Indeed, exhausting exercise involving stretch-shortening cycles has a more detrimental effect on the performance of these cycles themselves than on concentric performance, indicating that tolerance to repeated stretch declines as fatigue increases (Horita et al., 2003). In the case of purely eccentric exercise, impairment of muscle function following prolonged or exhaustive cycles of stretch-shortening has been proposed to arise from structural alterations, acute pro-inflammatory processes, accumulation of biochemical products and/or altered sensitivity of the stretch reflex and reduction in muscle stiffness during the eccentric phase of the exercise (Basmajian and De Luca, 1985; Sahlin, 1985; Green, 1997; Avela and Komi, 1998; Komi, 2000; Laaksonen et al., 2006).

### Cycle Length and the Duty Factor

The observation that cycle length is reduced on the second section while poling time remained unaltered here may reflect less total impulse during the poling action, i.e., a reduction in poling force. In the fatigued state, less effective dynamic contractions are due to slower development of force (Morel et al., 2015). In this context, an earlier report documented a detrimental effect of fatigue, induced by long-distance cll skiing, on the rate of force development by arm muscles (Boccia et al., 2016). Moreover, short-term, high-intensity skiing in the laboratory attenuates the ability to exert poling force significantly (Mikkola et al., 2013; Zoppirolli et al., 2016).

However, the poling force exerted during double poling by high-level cross-country skiers is related not only to muscle strength, but also depends on their technique, i.e., the expert use of body weight to transfer force to the pole. More specifically, the higher the forward lean of the body (i.e., the 𝜃COM) during the early poling phase, the higher the poling force (Zoppirolli et al., 2015, 2016). Although earlier studies showed little change in joint angles or displacement of the COM following maximal-intensity double poling of short duration (Zory et al., 2009; Zoppirolli et al., 2016), we found significant changes in the angles of the shoulder, elbow and ankle joints, as well as in the forward displacement of the COM relative to the feet (𝜃COM).

The reduction in the cycle length of elite cross-country skiers after 50 km of double poling racing can thus be interpreted as attenuated effectiveness of the poling action. This could be the result of lower capacity for propulsion through the poles due to muscle fatigue, reduced effectiveness of the stretch-shortening cycle of the arm extensor muscles, and/or less effective exploitation of body weight. Another important finding here was that, in agreement with the signs of progressive fatigue observed in connection with prolonged running (Vernillo et al., 2014; Giovanelli et al., 2017), the duty factor (i.e., the percentage of the cycle time accounted for by the poling phase) rose. Although cycle time was significantly lower on the second section, the duration of the poling phase was not reduced to the same extent, remaining similar to that on the first section (**Table 2**).

An elevated duty factor means relatively less time for muscle relaxation (Green, 1997). The higher percentage of the cycle time dedicated to propulsion probably reflects a reduced capacity of the arm extensor muscles to produce force rapidly. Boccia et al. (2016) suggested that the type of race examined here lowers the rate of voluntary force development by the elbow extensors, due to mechanical rather than neural reasons. At the same time, the shorter recovery phase means less blood flow through the muscles, possibly lowering ATP supplementation and the removal of metabolites (Green, 1997), as well as less time for repositioning the body properly for the subsequent propulsive phase.

Although the effects of utilizing double poling exclusively versus choosing a mixture of skiing techniques have never been compared systematically, our present findings reveal that racing for 2 h with only double poling alters whole-body kinematics and reduces effective propulsion.

## Methodological Considerations: Skiing On-Snow Versus on a Treadmill

In these final paragraphs, the reliability of our field-tracking methodology is assessed further by comparing our values for joint angles and displacement of the COM to those obtained by optoelectronic Motion Capture in laboratory studies. In particular the angles at the elbow, hip, knee and ankle joints on the first section here, when double poling velocity averaged 24.3 ± 0.9 km⋅h^-1^, can be compared to the findings of Holmberg et al. (2005) for double poling on roller skies at 24.5 ± 1.4 km⋅h^-1^ on a treadmill at a 1° incline. In addition, the displacements in *z*COM and 𝜃COM on the second section, when the mean double poling velocity was 22.5 ± 0.8 km⋅h^-1^, can be compared to our previous observations concerning double poling kinematics while roller skiing at 20.0 km⋅h^-1^ on a treadmill at a 1° incline (Zoppirolli et al., 2016).

All of the joints analyzed exhibited the typical flexion-extension pattern observed in the laboratory. More specifically, the elbow joint angle was approximately 80° at PP and 160° at PO, with maximal extension of about 170° after approximately 35% of the cycle time, both here on snow and in the laboratory (see **Table 3** and **Figure 5**, Holmberg et al., 2005). Even though the first phase of elbow joint flexion occupies approximately 10% of the cycle time in both these situations, the range of flexion was somewhat narrower on snow (with a minimum of 60° versus 50°, see **Figure 4**; Holmberg et al., 2005). In both situations as well, the hip joint angle was approximately 15° at PP, with maximal displacement of around 170° after 80% of the cycle time. Although this hip angle was minimal after 25% of the cycle in both cases, the range of flexion-extension was more pronounced on snow (a minimum of 80° versus 105° on a treadmill).

The displacements of the knee and ankle joints on snow were comparable to those in the laboratory only in a few portions of the cycle. Although the timing of flexion-extension is the same in both situations, the maximal angles of extension at both the knee (165°) and ankle (105°) after approximately 80% and 90% of the cycle time, respectively, were the only absolute angles that were similar. Indeed, when skiing on snow these joints are 10°–15° more extended during the rest of the cycle (see **Figure 4**, Holmberg et al., 2005).

Equally detailed comparison of the COM displacement is not possible, since Zoppirolli et al. (2016) assessed this parameter in the laboratory at only a few time-points during the DP cycle. When skiing on snow at approximately 22 km⋅h^-1^, the 𝜃COM here was about 8° at PP and -3° at PO, as was also the case on a treadmill. Moreover, the position of *z*COM (expressed as a percentage of body height) follows the same pattern in both situations, with comparable minimal and maximal values at PO and after 80% of the cycle, respectively (see **Table 3** and **Figure 5**; Zoppirolli et al., 2016).

In our opinion, these discrepancies between our findings and previous laboratory results concerning the displacement of joints and COM during a cycle of double poling reflect both differences in experimental conditions and the protocols employed. For example, the smaller range of flexion of the elbow joint on snow is probably due to the lower resistance of the snow than the treadmill to pole plant. At equal speed, double poling on snow should require less extensive stretch-shortening of arm extensors and, at the same time, put less stress on the shoulder and elbow joints. In contrast, the differences concerning the hip, knee and ankle joints can be ascribed to the different testing protocols, i.e., double poling on snow for several hours versus short-term double poling on a treadmill.

Overall, we conclude that our careful positioning of the video cameras, calibration of the tracks, choice of inclusion criteria and tracking procedures have provided reliable kinematic information. Indeed, both the displacement of joints and COM and the absolute values, pattern and timing of flexion-extension on the two sections filmed were highly similar to what has been observed in the laboratory. Experimental measurements in the field are challenging, especially with actual on-snow racing, with its changing environment, problems with instrumentation due to the temperature and reflections from the snow, lack of reference points for space calibration, etc. However, we demonstrate here that careful and rigorous methodology allows precise and reliable kinematic analysis, even under these difficult conditions.

## Conclusion

Racing for 2 h exclusively with double poling only affects the whole-body kinematics of elite cross-country skiers, even though they maintained their cycle frequency, irrespective of the actual velocity. Less effective positioning of the COM and altered flexion-extension of the elbow joint during the early portion of the poling phase were the major kinematic alterations observed. These changes, together with muscle fatigue, probably reduce the generation of poling force and thus cycle velocity with time. To minimize such detrimental effects, elite skiers should train maintaining optimal elbow and ankle kinematics and an efficient forward lean of the body during the propulsive phase of double poling, even when fatigued. As already mentioned, the technical training requires also specific focus on the strength and endurance of core, abdominal, and arm muscles.

## Ethics Statement

This study was pre-approved by the Ethical Committee of Verona University (prot. n° 5984/2015), as part of the project ”MarcialongaScience” proposed by the University of Verona and the Marcialonga Organizing Committee. When signing up for the race, the athletes gave their permission to be video-filmed. The present investigation contains data obtained from video-recordings.

## Author Contributions

CZ, BP, LB, and FSc contributed substantially to the conception and design of this study. CZ, BP, and LB contributed to data collection. CZ and FSt carried out the data analysis and interpretation together with BP, GB, and H-CH. CZ wrote the first draft of the manuscript and all authors were involved in revising it critically. CZ, BP, LB, FSt, GB, FSc, and H-CH gave final approval of the version to be published and agreed to be accountable for all aspects of this work.

## Conflict of Interest Statement

The authors declare that the research was conducted in the absence of any commercial or financial relationships that could be construed as a potential conflict of interest.The reviewer RF and handling Editor declared their shared affiliation at time of review.

## Footnotes

1 physlets.org/tracker
